# Ultrasound-Assisted Green Natural Deep Eutectic Solvent Extraction of Flavonoids from Wild Blueberry: Process Optimization, Composition Identification, and Antioxidant Activity

**DOI:** 10.3390/foods14193325

**Published:** 2025-09-25

**Authors:** Le Ouyang, Weiwei Liang, Chun Bian, Yi Shan, Shumei Wang

**Affiliations:** 1 College of Food Engineering, Harbin University, Harbin 150086, China; oyl2022@hrbu.edu.cn (L.O.); bianchun1980@126.com (C.B.); shanyi1987hlj@163.com (Y.S.); wangshumei@126.com (S.W.); 2 College of Food Science, Northeast Agricultural University, Harbin 150030, China

**Keywords:** wild blueberry flavonoids, ultrasonic-assisted, natural deep eutectic solvent, component analysis, antioxidant activity

## Abstract

To improve the deep processing and utilization of wild blueberries, this study presents a green and highly efficient method for extracting flavonoids from blueberries. The approach combines natural deep eutectic solvents (NADESs) with ultrasound-assisted extraction. Among the 22 tested NADES, Betaine/urea (BU), was the most effective solvent for extracting flavonoids from blueberries. The extraction parameters of ultrasound-assisted betaine/urea (UABU) were optimized using a response surface methodology (RSM). This optimization procedure yielded the optimized conditions outlined below: a molar ratio of urea to betaine of 3.3:1, a water content of 60% (*m*/*v*), an ultrasonic power of 330 W, a solid-to-liquid ratio of 1:30, an extraction temperature of 50 °C, and an ultrasonic extraction duration of 30 min. Under these conditions, the total flavonoid content (TFC) extracted using UABU reached 6.06 ± 0.024 mg_RE/g_DW, a 1.44-fold increase compared to ultrasound-assisted 70% (*v*/*v*) ethanol (UAE). Liquid Chromatography–Tandem Mass Spectrometry (LC-MS/MS) nontargeted metabolomics analysis revealed that the flavonoids extracted by UABU had highly relative content (RC) of Oenin, 3′-methoxy-4′,5,7-trihydroxyflavonol, Isorhamnetin-3-O-glucoside and Isoquercitrin. Significant disparities exist regarding the types and RC of flavonoids obtained via UAE. Results from in vitro antioxidant assays demonstrated that UABU has superior antioxidant activity relative to UAE. This study demonstrated the feasibility of using NADESs, specifically BU, as an efficient and eco-friendly extraction medium for flavonoids from wild blueberries. The yield of flavonoids was increased by this method, and bioactive compounds were also protected—findings that underscore the potential of green solvents for application in the food industry.

## 1. Introduction

Blueberry, a perennial shrub and small berry-bearing tree belonging to the genus Vaccinium, enjoys global popularity due to its high nutritional and health benefits. Rich in anthocyanins, flavonoids, phenolic acids, vitamin compounds, and other substances, it serves as an abundant source of bioactive components in the diet [1]. As a result, it has been selected as one of the five health foods for humans by the Food and Agriculture Organization of the United Nations [2]. Flavonoids mainly include flavones, flavonols, flavanones, flavanols, flavanonols, dihydroflavonols, and a variety of other flavonoid derivatives [3]. These flavonoids exhibit significant antioxidant [4], vision improvement [5], anti-inflammatory [6], and cardiovascular disease prevention properties [7]. At present, the main methods for extracting blueberry flavonoids compounds include distillation method, microwave-assisted extraction method, ultrasound-assisted extraction method, microjet extraction method, flash extraction method, etc. The aforementioned methods commonly employ traditional organic solvents (methanol, ethanol, acetone, and chloroform) as extraction agents. However, traditional organic solvents have issues such as being volatile, having poor safety, and posing certain toxicity to the human body [8]. Therefore, in-depth research and optimization of the extraction solvents for flavonoids compounds have become a more noteworthy issue at present.

In recent years, deep eutectic solvents (DESs) have been widely studied and applied in the extraction of functional components from plants, which represent a new class of solvents composed of hydrogen bond donors (HBDs) and hydrogen bond acceptors (HBAs). These solvents form a mixture that has a melting point lower than the individual components. DESs address the drawbacks of traditional organic solvents, such as high toxicity, volatility, flammability, and explosiveness. They have established themselves as highly effective and environmentally benign green solvents, boasting benefits including low melting points, excellent thermal and chemical stability, as well as low volatility [9]. Furthermore, the hydroxyl or carboxyl groups contained in DES constituents form hydrogen bonds with target compounds, thereby boosting both the extraction efficiency and the stability of these compounds [10]. Compared to ionic liquids, another class of green solvents, DESs maintain high extraction efficiency and are easily degradable. Moreover, they avoid drawbacks such as residue accumulation, and high costs [11,12,13,14,15]. Among DESs, natural deep eutectic solvents (NADESs), composed of natural components like primary metabolites, are particularly noteworthy. NADESs can facilitate more efficient extraction of bioactive ingredients while preserving their stability and safety. Due to their environmentally friendly properties, such as biodegradability and reusability, NADESs are not only effective for extracting flavonoids compounds outperforming other DESs in this regard [16,17,18,19]. Moreover, the ultrasonic extraction method utilizes high-frequency sound waves to generate cavitation and perturbation effects, thereby enhancing the efficiency of mass transfer. It is also widely applied in the extraction of plant flavonoids compounds due to its simple operation, short cycle time, low cost, and good stability.

Cheng et al. [20] evaluated 22 distinct types of DES for ultrasonically extracting flavonoids from peanut leaves and stems. Out of these, choline chloride-acetic acid (ChCl-Aa) with a molar ratio of 1:2 demonstrated superior efficacy in comparison to other solvents. The optimal extraction parameters were established as follows: a DESs/H_2_O mixture containing 27% (mol%) water, an extraction duration of 43 min, and a liquid-to-solid ratio of 31:1 g/mL. Under these conditions, the RSM yielded a flavonoid concentration of 2.980 mg/g DW. Zhang et al. [21] succeeded in synthesizing ten varieties of NADESs, and these solvents were then employed in combination with microwave-assisted extraction (MAE) to extract total flavonoid compounds from discarded sweet potato (*Ipomoea batatas* L.) leaves. Through single factor experiments and response surface methodology (RSM) optimization, the NADESs synthesized from choline chloride and malic acid (molar ratio 1:2) exhibited the highest extraction yield. In vitro bioactivity experiments confirmed that total flavonoid compounds exhibited strong 1,1-Diphenyl-2-picrylhydrazyl (DPPH) and O_2_^−^ radical-scavenging activity, along with inhibitory properties against *Escherichia coli*, *Staphylococcus aureus*, and *Bacillus subtilis*. Previous studies have shown that ultrasound-assisted NADESs extraction (UANE) represents an environmentally friendly approach for extracting active ingredients from natural resources and warrants further development. However, although DESs has demonstrated good research value and promising prospects in the extraction of active substances, its current application is still at the “trial-and-error” stage, necessitating systematic and in-depth research to clarify the conditions for its application.

Therefore, this study aims at the efficient extraction of flavonoid compounds from wild blueberries. It screens out the NADESs with the optimal composition and component ratio as the extraction reagent. Through single-factor experiments and RSM optimization, it explores the best extraction process for flavonoid compounds from blueberries. Using traditional organic solvent (ethanol) as a control, it analyzes the component composition and antioxidant capacity of flavonoid compounds extracted by different solvents to verify the effectiveness of NADESs as an extraction solvent. This provides a feasible basis for research on replacing traditional organic solvents with NADESs for the extraction of natural products and offers data support for further enhancing the added value of wild blueberry products.

## 2. Materials and Methods

### 2.1. Materials and Reagents

Fresh wild blueberries were sourced from a local farmers’ market in Mohe City, Greater Khingan Mountains Prefecture, Heilongjiang Province, China, and stored at −18 °C. Choline chloride and oxalic acid were sourced from Macklin Biochemical Co., Ltd., located in Shanghai, China. Betaine and rutin (analytical standard, HPLC ≥ 98%) were obtained from Yuanye Bio-Technology Co., Ltd. (Shanghai, China). Sorbitol was purchased from Guangfu Institute of Fine Chemicals (Tianjin, China). Ethylene glycol and absolute ethanol were obtained by Fuyu Fine Chemical Co., Ltd. (Tianjin, China). Glycerol and urea were obtained from Tianli Chemical Reagent Co., Ltd. (Tianjin, China). Additionally, 1,4-butanediol, DL-malic acid and tartaric acid were sourced from Fuchen Chemical Reagent Co., Ltd. (Tianjin, China), and citric acid, aluminum nitrate, and hydrochloric acid were obtained from Kemiou Chemical Reagent Co., Ltd. (Tianjin, China). Lactic acid was purchased from Hengxing Chemical Reagent Manufacturing Co., Ltd. (Tianjin, China), and L-proline was supplied by Lanji Biotechnology Co., Ltd. (Shanghai, China). L-ascorbic acid, sodium nitrite and sodium hydroxide were obtained from Sinopharm Chemical Reagent Co., Ltd. AB-8 macroporous adsorption resin was sourced from Huida Chemical Co., Ltd. (Tianjin, China). Acetonitrile and methanol were purchased from Merck (Darmstadt, Germany). Formic acid was purchased from Anpel Experimental Technology Co., Ltd. (Shanghai, China). The ABTS free radical scavenging assay kit was purchased from Solarbio Science & Technology Co., Ltd. (Beijing, China). The hydroxyl radical scavenging assay kit (Fenton colorimetric method) was sourced from Yuanye Bio-Technology Co., Ltd. (Shanghai, China), and the 1,1-diphenyl-2-picrylhydrazyl radical (DPPH) scavenging assay kit was acquired from Jiancheng Bioengineering Institute (Nanjing, China).

### 2.2. Preparation of NADESs

Based on previous research, choline chloride and betaine were selected as HBA for the experimental investigation. Additionally, a range of representative substances was chosen as HBDs, including alcohols (sorbitol, ethylene glycol, propylene glycol, and butanediol), organic acids (citric acid, malic acid, oxalic acid, lactic acid, and tartaric acid), amide (urea) and amino acid (proline). A total of 22 types (Table 1) of NADESs were prepared by mixing the selected HBAs and HBDs in appropriate molar ratios with deionized water (wt%) to achieve the desired blends. The mixtures were continuously stirred at 300 r/min at 80 °C until a transparent and homogeneous liquid was obtained [15].

### 2.3. Screening of NADESs

The screening of NADESs was conducted using conventional heat-assisted extraction. Thawed wild blueberries were ground into a slurry using a mortar at 4 °C. A 1.0 g sample of blueberry puree was added to 20 mL of the pre-prepared NADESs as shown in Table 1.The mixture was then placed in an HH-8A digital constant-temperature magnetic stirring water bath (Changzhou Ronghua Instrument Manufacturing Co., Ltd., Changzhou, China), heated to 50 °C, and stirred at 300 r/min for 3 h. Following extraction, the supernatant was obtained via centrifugation (TD5A-WS high-speed centrifuge, Hunan Xiangyi Laboratory Instrument Development Co., Ltd., Changsha, China) at 3000× *g* for 10 min. The collected supernatant was subsequently analyzed to determine the total flavonoid content (TFC: the TFC values obtained via this method may include contributions from phenolic acids and may underestimate the content of flavonoids compounds without a catechol moiety (e.g., isorhamnetin, kaempferol) due to variable absorption coefficients among different flavonoids) [22,23]. Each extraction condition was replicated three times, and the average value was determined.

### 2.4. Extraction and Determinationof Total Flavonoids from Blueberries

A 1.0 g sample of blueberry puree was precisely weighed out and transferred into a 250 mL flask. The pre-chosen NADESs were added to the flask in line with the pre-set solvent-to-solid ratio. The flask was then placed in a JP-180st bath-type ultrasonic instrument (Shenzhen Jiemeng Technology Co., Ltd., Shenzhen, China; Tank Capacity: 53 L, Heater: 0–80 °C adjustable), which has an adjustable ultrasonic power range of 0–900 W and operates at an ultrasonic frequency of 40 kHz. After undergoing ultrasonication for a designated time period, the extract was moved to a high-speed centrifuge and centrifuged at 3000× *g* for 10 min at a temperature of 25 °C. The supernatant was gathered for subsequent testing. Each extraction condition was repeated three times to guarantee the reliability of results, with the average value being documented.

**Figure 1:**
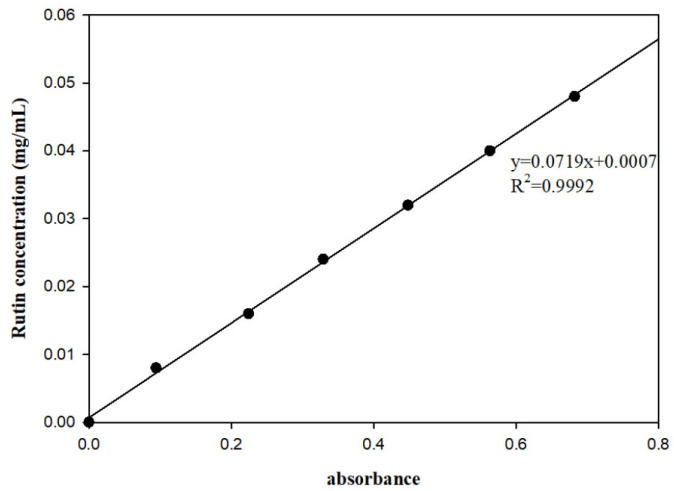
Standard curve for rutin.

TFC was assayed using the colorimetric method relying on the sodium nitrite-aluminum nitrate-sodium hydroxide (NaNO_2_–Al (NO_3_)_3_–NaOH) system [24,25], with slight adjustments, and rutin was employed as the standard substance. A precisely weighed 5 mg sample of rutin was dissolved in a 25 mL volumetric flask containing a 50% (*v*/*v*) ethanol solution to prepare a stock solution. Then, 1.0, 2.0, 3.0, 4.0, 5.0, and 6.0 mL aliquots of the stock solution (still containing NADES, without column purification or lyophilization) were dispensed into individual 25 mL measuring flasks. Each aliquot of the solution was combined with 1 mL of a 5% (*w*/*v*) sodium nitrite solution and allowed to react at a temperature of 22 ± 2 °C for a duration of 6 min. Subsequently, an additional 1 mL of a 10% aluminum nitrate solution was introduced, allowing the reaction to proceed for a further 6 min. Ultimately, 10 mL of a 4% (*w*/*v*) sodium hydroxide solution was added to the mixture. The reaction solution was diluted to a total volume of 25 mL with 50% (*v*/*v*) ethanol, followed by thorough mixing and a 15 min incubation period at 22 ± 2 °C. Following high-speed centrifugation for 6 min (3000× *g* and 25 °C), the absorbance of the supernatant was measured at 510 nm using a T6 New Century UV Visible Spectrophotometer (Beijing Puxi General Instrument Co., Ltd., Beijing, China). The TFC in blueberries was determined by applying the subsequent formula: *TFC* (mg/g) = *C* × N × *V*/*M*, where C represents the concentration obtained by inputting the measured absorbance into the standard curve (Figure 1.,mg/mL), N represents the dilution factor, V is the total volume of the extraction solution (mL), and M is the weight of the blueberry sample. All measurements were performed in triplicate, and the mean values were calculated.

### 2.5. Optimization of UANE Conditions for Total Flavonoid Content

#### 2.5.1. Single Factor Experiments Design

Single-factor tests were carried out, wherein only one key influencing factor was adjusted whereas all other conditions were maintained unchanged. The objective of these experiments was to optimize six parameters: the molar ratio of HBAs to HBDs (A: 3:1, 2:1, 1:1, 1:2, 1:3, and 1:4), water content in the NADESs system (B: 30, 40, 50, 60, 70, and 80%), ultrasonic power (C: 0, 150, 300, 450, 600, and 750 W), extraction time (D: 10, 15, 20, 25, 30, and 35 min), extraction temperature (E: 35, 40, 45, 50, 55, and 60 °C), and solid–liquid ratio (F: 1:10, 1:20, 1:30, 1:40, 1:50, and 1:60 g/mL). TFC was used as the response variable. Factors showing a significant impact on TFC were selected for response surface experiments [26,27].

#### 2.5.2. Response Surface Methodology Experiments

Drawing on the results of single-factor tests, an experiment using response surface methodology (RSM) that adopted a three-factor-three-level Box–Behnken Design (BBD) was conducted [28]. The purpose was to study how different variables influence TFC and evaluate the interactions among these factors. Three independent variables were selected: the molar ratio of HBDs to HBAs (A), the water content of the NADESs system (B), and ultrasonic power (C). TFC was considered the response variable (Y). A 3D graph-based analysis was performed using the Design Expert 13.0 software (Stat-Ease, Inc., Minneapolis, MN, USA). In order to confirm the accuracy and dependability of the model, the experimental values obtained under the optimal conditions were compared against the predictions of the model.

### 2.6. Identification of Main Flavonoids Relative Content Using LC-MS/MS Untargeted Metabolomics

Under identical process parameters (including ultrasonic time, ultrasonic power, ultrasonic temperature, and solid-to-liquid ratio), blueberry flavonoids were extracted using the selected NADES and 70% ethanol (as a control), respectively. The main flavonoids were separated and purified prior to LC-MS/MS analysis. AB-8 microporous resins were pretreated according to the procedure described by Yang et al. [29], with minor modifications. The resins were immersed in 95% (*v*/*v*) ethanol for a period of 24 h to facilitate complete swelling. Subsequently, the swollen resins were packed into the column using wet methods and dynamically rinsed with 95% (*v*/*v*) ethanol, followed by distilled water to remove residual alcohol. After being soaked in 4 times the volume of 5% HCl (*v*/*v*) solutions for 5 h, The swollen resins were rinsed with distilled water until they attained a neutral pH level. Then the swollen resins were rinsed with distilled water until they attained a neutral pH level, after being soaked in 4 times the volume of 5% NaOH (*w*/*v*) solution for 5 h. The height-to-diameter ratio of the chromatography column was 1:10 and the column volume was 2.2 L. The sample volume was 1/4 of the column volume, and the sample loading rate was 1.0 mL/min. After static adsorption for 2 h, the chromatography column was rinsed with distilled water at twice the column volume. The eluent was a 65% (*v*/*v*) ethanol solution, and the elution rate was 2.0 mL/min. The purified extract was then collected, rotary evaporated, freeze-dried, and weighed.

The freeze-dried sample was placed into a pre-cooled grinding chamber (precooled to −20 °C), and 5 mm-diameter stainless steel balls were added to the chamber. The grinder was set to an agitation speed of 300 rpm, and the sample was subjected to one single 10 min grinding run to convert it into a powdered form. After grinding, the resulting powder was passed through an 80-mesh sieve to ensure uniform particle size. Subsequently, 50 mg aliquot of the powdered sample was combined with 500 µL of a 75% (*v*/*v*) methanol-water solution and exposed to ultrasonic treatment (300 W, 20 kHz, 25 ± 2 °C) for a duration of 30 min. Following this, the mixture was centrifuged at a temperature of 20 °C and a rotational speed of 17,000× *g* (*relative centrifugal force*) for a period of 10 min. The supernatant obtained was then transferred to an injection vial, prepared for subsequent analysis.

An LC-MS/MS system, equipped with an H-class ultra-high pressure liquid phase chromatograph (Waters, Milford, MA, USA), was used for the analysis. The system included an ACQUITY UPLC HSS T3 column (1.8 μm, 2.1 × 100 mm; Waters, Milford, MA, USA) and 6600 a QTOF high-resolution mass spectrometer (AB SCIEX, Framingham, MA, USA). The mobile phase comprised two components: 0.1% (*w*/*v*) formic acid aqueous solution (A) and 0.1% (*w*/*v*) formic acid in acetonitrile (B). The elution gradient profile is detailed in Table 2. The analytical parameters encompassed a 40 °C temperature and a 40 μL injection volume. The AB 6600 Triple TOF mass spectrometer acquired primary and secondary mass spectral data via the Data-Dependent Acquisition (IDA) function regulated by Analyst TF 1.7 software (AB Sciex). In each data acquisition cycle, the mass spectrometer selected molecular ions with the highest intensity (exceeding 100) to collect the corresponding secondary mass spectral data. The first-stage acquisition range was 50–1200 *m*/*z*, the collision energy was set to 30 eV, and 15 s level spectra were collected per cycle. Parameters for the ESI ion source were set as follows: atomization pressure (GS1) set to 60 psi, auxiliary gas pressure of 60 psi, curtain gas pressure of 35 psi, a 550 °C temperature, and a spray voltage of 5500 V (in positive ion mode) or −4500 V (in negative ion mode).

The MSDIAL software, version 4.6, was utilized for peak detection, peak alignment, and comprehensive data processing of the converted ABF files. The identification outcomes were derived from the analysis of both primary and secondary mass spectra, coupled with independent integrations referencing databases (version 6.0) including Metlin, MassBank, MoNA, and HMDB. The parameter settings for the MSDIAL software specified an accurate mass tolerance of 0.01 Da for MS1, 0.05 Da for MS2, and an identification score threshold set at 60%.

The relative quantitative analysis was calculated from the peak area of identified compounds [30]. The RC was determined using the equation provided below.

$${\mathrm{RC}}_{i}=A_{j}/\sum_{j=1}^{n}A_{j}\times \mathrm{100}\%$$

where *RC_i_* represents the RC of the i-th flavonoid component (%), *A_i_* represents the peak area of the i-th flavonoid component obtained from the LC-MS/MS analysis, $\sum_{j=1}^{n}A_{j}\;$ represents the sum of the peak areas of all the “n” detected components.

### 2.7. FT-IR Spectroscopy and Differential Scanning Calorimeter (DSC)

The architectural features of the freeze-dried specimen were probed through FT-IR analysis, employing a Nicolet IS50 Spectrometer sourced from Thermo Scientific in the Waltham, MA, USA. The lyophilized phenolic compounds, which had been synthesized utilizing the column-based purification approach delineated in Section 2.6, were integrated into KBr pellets. These pellets were then subjected to scanning across a wavenumber spectrum spanning from 4000 to 400 cm^−1^.

Regarding the assessment of the freeze-dried sample’s thermal resilience, DSC examination was carried out using a DSC200 instrument manufactured by Hitachi in Tokyo, Japan. The lyophilized dried specimens were positioned within a capped aluminum crucible. Subsequently, they were heated under a nitrogen atmosphere, with the temperature incrementing from 30 °C to 152 °C at a steady pace of 10 °C per minute.

### 2.8. In Vitro Antioxidant Activity

#### 2.8.1. Hydroxyl Radical Scavenging Capacity

The scavenging ability of hydroxyl radicals was assessed utilizing an assay kit specifically designed for hydroxyl radical antioxidants, sourced from Yuanye Bio-Technology Co., Ltd. (Shanghai, China). The kit, which was used in accordance with the provided manual, contained the following reagents: (a) phenanthroline solution, (b) OH assay buffer, (c) ferrous chromogenic solution, and (d) oxidant. (The kit detection principle is as follows: H_2_O_2_/Fe^2+^ generates hydroxyl radicals through the Fenton reaction, and Fe^2+^ is oxidized to Fe^3+^ in this process. This oxidation reaction causes the red 1,10-phenanthroline-Fe^2+^ complex to be converted into the colorless 1,10-phenanthroline-Fe^3+^ complex, leading to the disappearance of the maximum absorption peak of 1,10-phenanthroline-Fe^2+^ at 536 nm. The change in absorbance within the wavelength range of 530–540 nm can be measured using a microplate reader; based on this change, the variation in hydroxyl radical content can be calculated, and thus the hydroxyl radical scavenging rate or scavenging capacity of the sample can be determined). Absorbance was measured using the T6 New Century UV-visible spectrophotometer. The reaction mixture was formulated by mixing the working solution with the sample solution or ascorbic acid solution. The optical density was measured at 535 nm after incubating the mixture in a water bath at 37 °C for 1 h [31]. The scavenging rate of hydroxyl radicals was determined using the equation provided below.

•OH scavenging rate (%) = [(A_3_ − A_3′_) − (A_2_ − A_0_)]/(A_1_ − A_2_) × 100%

A_0_ = absorbance of blank tube; A_1_ = absorbance of positive control tube; A_2_ = absorbance of negative control tube; A_3′_ = absorbance of control tube; A_3_ = absorbance of extract or ascorbic acid solution tube. All tests were conducted in triplicate to ensure the calculation of a mean value.

#### 2.8.2. DPPH Radical Scavenging Activity Measurements

The DPPH radical scavenging activity was evaluated using the DPPH antioxidant assay kit obtained from Jiancheng Bioengineering Institute (Nanjing, China), and the kit was used in accordance with the manual provided. Absorbance was measured using the T6 New Century UV-visible spectrophotometer. The reaction mixture was formulated by mixing the DPPH working solution and 80% (*v*/*v*) methanol with the sample solution or ascorbic acid solution (as positive control). According to the kit instructions, 0.4  mL of sample solution was mixed with 0.6 mL of DPPH· working solution and allowed to react in the dark at room temperature (23 °C  ±  2 °C) for 30 min followed by centrifugation at 3000× *g* for 5 min, after which the absorbance was measured at 517 nm. An equal volume of 80% (*v*/*v*) ethanol instead of the sample solution was used as the control, and an equal volume of 80% (*v*/*v*) ethanol instead of DPPH· working solution was used as the sample blank. Ascorbic acid (0, 5, 10, 15, 20, 25  μg/mL) was used as a positive control [32]. The free-radical scavenging activity of DPPH was calculated using the following equation:

DPPH radical scavenging (%) = (1 − (A*_Sample_* − A*_control_*)/A*_blank_*) × 100%

where *A_Sample_* represents the absorbance of the extract or ascorbic acid reacting with DPPH working solution, *A_control_* represents the absorbance of the extract or ascorbic acid mixed with 80% (*v*/*v*) methanol, *A_blank_* represents the absorbance of the mixture of DPPH working solution and 80% (*v*/*v*) methanol. All tests were conducted in triplicate to ensure the calculation of a mean value.

#### 2.8.3. ABTS Radical Scavenging Activity Assay

The ABTS radical scavenging activity was assessed via the ABTS antioxidant assay kit provided by Solarbio Science & Technology Co., Ltd. (Beijing, China), in strict accordance with the procedure specified in the kit manual. A mixture of 50 µL of the sample solution and 850 µL of ABTS solution was prepared, and the reaction was conducted in the dark for 6 min at 23 ± 2 °C. The absorbance of the mixture was then measured at a wavelength of 405 nm. For the blank control, ultrapure water was used in place of the sample solution. Ascorbic acid solution served as the positive control by replacing the sample solution. A control tube containing only the sample solution was also included [33]. The ABTS radical scavenging rate (D%) was calculated via the formula presented below:

D_sample_ (%) = [A*_blank_* − (A*_sample_* − A*_control_*)]/A*_blank_* × 100%

DAscorbic acid (%) = (A*_blank_* − A*_positive_*-*_controlled_*)/A*_blank_* × 100%

where *A_blank_* represents the absorbance of the blank tube, *A_sample_* represents the absorbance of the extract solution tube, *A_control_* represents the absorbance of the control tube, and *A_positive_-_controlled_* represents the absorbance of the ascorbic acid solution tube. All tests were conducted in triplicate to ensure the calculation of a mean value.

### 2.9. Statistical Analysis

To assess the differences across various treatment groups, a one-way analysis of variance (ANOVA) was carried out utilizing IBM SPSS Statistics software, version 26.0 (developed by SPSS, Inc., located in Chicago, IL, USA). After that, Duncan’s multiple range test (post hoc test) was applied. Treatment differences were deemed statistically significant when the least significant difference values indicated a probability level of *p* < 0.05. The response surface experiment was performed using Design Expert 13.0 software (Stat-Ease, Inc., Minneapolis, MN, USA). Sigma Plot 12.5 software was used for dynamic analysis and graph plotting. The data obtained from all triplicate experiments is presented as the mean value accompanied by the standard deviation.

## 3. Results and Discussion

### 3.1. Selection of NADESs for the Extraction of Total Flavonoid Content from Wild Blueberries

**Figure 2:**
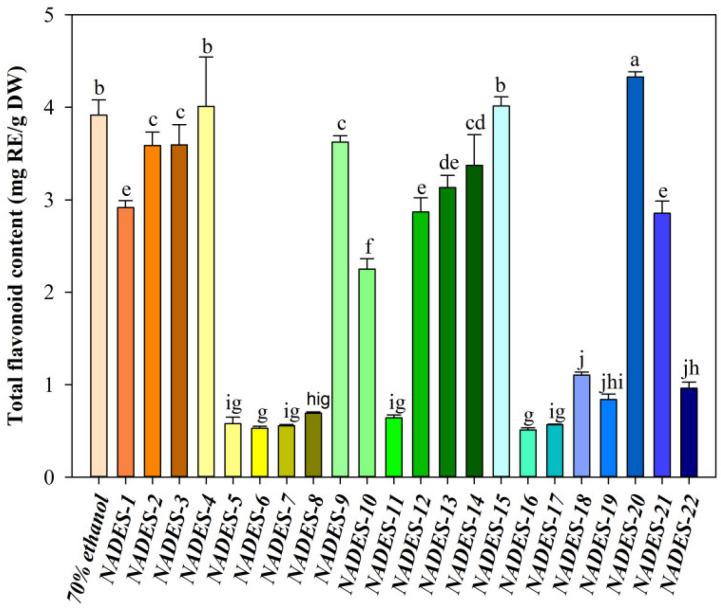
Effect of natural deep eutectic solvent (NADES) types on total flavonoid content of blueberry extracts derived from the same batch of berries. Different letters indicate significant differences between groups at *p* < 0.05.

The efficiency of extraction is predominantly determined by the polarity and viscosity of NADESs. These properties are significantly influenced by multiple factors, which include the specific makeup of the NADESs, the molar ratio of HBA to HBD, and the water content in the solvent system [34]. The formation and stability of the eutectic system are largely governed by the specific composition of NADESs. Research has shown that the efficient extraction of total flavonoids can be notably improved when utilizing NADESs. Such NADESs are prepared using HBAs like choline chloride or betaine, along with HBDs such as polyols, organic acids, sugars, or amides [35]. We chose choline chloride and betaine as HBAs, both of which are from natural plant materials, cost-effective, and readily accessible. Sorbitol, ethylene glycol, propylene glycol, butanediol, citric acid, malic acid, oxalic acid, lactic acid, tartaric acid, urea, and proline were selected as HBDs to prepare 22 NADESs. The analysis indicated notable variations in the TFC extracted, depending on the type of NADESs (Figure 2). The maximum TFC extraction yield was obtained with BU (4.33 ± 0.06 mg_RE/g_DW), which was significantly higher than the TFC extracted using 70% (*v*/*v*) ethanol (3.92 ± 0.16 mg_RE/g_DW, *p* < 0.05), followed by Choline chloride/Butanediol (4.01 ± 0.53 mg_RE/g_DW) and Betaine/Butanediol (4.01 ± 0.10 mg_RE/g_DW).

In addition to the three NADES solutions mentioned above, the TFC obtained from the remaining NADES solutions was significantly lower than the TFC extracted using 70% (*v*/*v*) ethanol (*p* < 0.05, Figure 2). The enhanced dissolution of blueberry flavonoids in BU can primarily be attributed to the polarity match between the solvent and solute, The moderate viscosity of BU ensures efficient mass transfer during extraction, without causing excessive hindrance to diffusion [36]. In this research, BU was chosen as NADES because it had the highest extraction rate. Moreover, the viscosity of BU is appropriate, which is helpful for the precipitation of TFC. This finding is consistent with the results reported by Peng et al. [16]. They investigated the effect of 8 NADESs on the extraction of flavonoids from *Moringa oleifera* Lam. Leaves. The experimental results verified that the extraction yield of ultrasound-assisted NADES composed of betaine and urea had the highest extraction rate, reaching 54.69 ± 0.19 mg_RE/g_DW. As a result, BU was chosen as the best green solvent for the following experiments.

### 3.2. Single Factor Experiments

**Figure 3:**
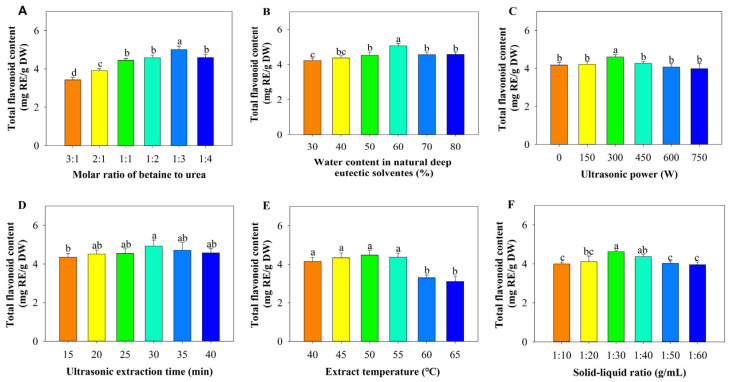
Effect of ultrasound-assisted betaine-urea extraction on total flavonoid content: (**A**) Molar ratio of betaine to urea, other parameters ((**B**): 30%, (**C**): 300 W, (**D**): 20 min, (**E**): 45 °C, (**F**): 1:20)); (**B**) Water content in natural deep eutectic solvents, other parameters ((**A**): 1:1, (**C**): 300 W, (**D**): 20 min, (**E**): 45 °C, (**F**): 1:20)); (**C**) Ultrasonic power, other parameters ((**A**): 1:1, (**B**): 30%, (**D**): 20 min, (**E**): 45 °C, (**F**): 1:20)); (**D**) Ultrasound extraction time, other parameters ((**A**): 1:1, (**B**): 30%, (**C**): 300 W, (**E**): 45 °C, (**F**): 1:20)); (**E**) Extraction temperature, other parameters ((**A**): 1:1, (**B**): 30%, (**C**): 300 W, (**D**): 20 min, (**F**): 1:20)); (**F**) Solid–liquid ratio, other parameters ((**A**): 1:1, (**B**): 30%, (**C**): 300 W, (**D**): 20 min, (**E**): 45 °C)). Different letters indicate significant differences between groups at *p* < 0.05.

Based on the results in Section 2.1, BU was chosen as the NADES for extracting blueberry total flavonoids. To determine an optimal NADESs system, the molar ratio between BU was varied, as well as the solvent content, on the extraction efficiency were systematically examined. The TFC initially increased and then decreased as the molar ratio of betaine to urea was increased (Figure 3A). The maximum extraction yield (5.01 ± 0.17 mg_RE/g_DW) was achieved at a 1:3 (betaine–urea) molar ratio, which was significantly higher (*p* < 0.05) than yields at other ratios. Prior research has consistently highlighted the significance of the molar ratio between HBAs and HBDs in NADESs for the extraction process. Achieving an optimal ratio optimizes the interaction between the HBA and HBD components. However, excessively high molar ratios can result in elevated viscosity, which in turn diminishes the solvent’s capacity to dissolve the extract [37]. Concurrently, decreased viscosity and increased polarity [38], along with an increased contact surface area with the material and an elevated conductivity rate, enhance the dissolution rate of total flavonoids. The impact of water content on TFC was evaluated across a gradient of 30–80% (wt%, Figure 3B). The highest yield (5.06 ± 0.13 mg_RE/g_DW) was obtained at 60% water addition, which was significantly superior (*p* < 0.05) to yields at 30% (3.82 ± 0.11 mg RE/g DW). The observed decrease can be ascribed to the effect where water content decreases viscosity and elevates polarity, leading to the disruption of hydrogen bonds among molecules and consequently disturbing the supramolecular architecture of the system [39]. Consequently, the weakening of interactions between the solvent and the flavonoids leads to a decrease in TFC.

A series of single-factor experiments were performed to assess the impact of ultrasonic power and ultrasound extraction time on the TFC. The robust cavitation and mechanical vibrations generated by ultrasound can disrupt the plant cell structure, leading to the rapid dissolution of target total flavonoids from wild blueberry cells [40]. As shown in Figure 3C, TFC exhibited a dose-dependent increase with ultrasonic power up to 300 W, peaking at 4.60 ± 0.12 mg_RE/g_DW, which was significantly higher (*p* < 0.05) than yields at other power. This phenomenon can be explained by the enhanced molecular motion and improved cell wall disruption facilitated by higher ultrasonic energy, which promotes the release of flavonoids [41]. Nevertheless, higher ultrasound power does not consistently enhance extraction efficiency, as an excessive amount of ultrasonic energy can disrupt the structural integrity of flavonoids, leading to a reduction in the overall yield of flavonoid extraction [42]. Therefore, when the ultrasonic power was further increased, the opposite trend was observed. Overly high ultrasonic power can intensify cavitation effects, resulting in the production of an increased number of impurities and the breakdown of flavonoid compounds, which ultimately diminishes the overall extraction efficiency [43]. However, the degradation products of flavonoid compounds were not examined in this study. In addition, ultrasound treatment generates heat through two primary mechanisms. First, cavitation effects occur when microbubbles collapse during ultrasonic exposure, releasing localized energy that elevates the bulk temperature of the system. Second, viscous dissipation takes place as mechanical energy is converted to heat through molecular friction within the liquid medium. Consequently, at higher ultrasound powers, the actual temperature may exceed the optimal range, potentially reducing TFC [44].

Similarly, the duration of ultrasound treatment also plays a significant role in the extraction process. The impact of time on the extraction rate of TFC was investigated over a duration ranging from 15 to 40 min, while maintaining all other experimental conditions constant. When the ultrasonic time increased from 15 min to 30 min, the extraction yield increased proportionally (Figure 3D). The TFC reached its highest value of 4.93 ± 0.31 mg_RE/g_DW at 30 min of ultrasonic treatment. This phenomenon can be attributed to the cavitation effect of ultrasonic energy, which breaks down cell walls and facilitates the release of flavonoids [45]. However, the TFC had no significant difference (*p* > 0.05) between 30 min and 40 min treatments. This suggests that 30 min suffices for complete cell disruption and flavonoid release under optimized power conditions (300 W).

To assess the effects of extraction temperature and solid–liquid ratio on TFC, single-factor experiments were conducted the temperature range 45 to 65 °C and the solid–liquid ratio range of 1:10 to 1:60. The TFC increased from 4.15 ± 0.22 mg_RE/g_DW at 45 °C to 4.48 ± 0.24 mg_RE/g_DW at 50 °C. Nevertheless, when the temperature exceeded 50 °C, a significant decline (*p* < 0.05) in TFC was observed (Figure 3E). This trend can likely be explained by the reduction in solvent viscosity at elevated temperatures, which diminishes the energy threshold necessary for effective extraction and cavitation generation [46]. Nevertheless, temperatures surpassing 50 °C may accelerate the degradation of flavonoids, counteracting the enhanced mass transfer typically associated with higher thermal conditions [47].

With the solid–liquid ratio ranging from 1:10 to 1:30, there was a significant rise in the extraction yield (*p* < 0.05), which was increased from 4.00 ± 0.09 to 4.63 ± 0.06 mg_RE/g_DW. The increase in extraction efficiency can be attributed to the enlarged interface between the blueberry puree and the solvent, which promotes a more efficient extraction process and a continuous release of total flavonoids [16]. However, when the solid–liquid ratio exceeded 1:40, the TFC began to significantly decrease (*p* < 0.05). This is possibly due to reduced mixture density, which leads to faster ultrasound wave propagation. Consequently, this reduces the ultrasound power attenuation effect and increases energy transfer, potentially leading to the thermal degradation of bioactive compounds and resulting in a reduction in the TFC [48]. In addition, at higher solvent volumes, the increased surface area of the solvent–flask interface may enhance the non-specific adsorption of target compounds onto glass surfaces. Extract residues may adhere to the flask walls, thereby reducing the amount available for spectrophotometric quantification. Excessive solvent may dilute the extract below the detection limit of the spectrophotometric method, leading to an underestimation of yield [41]. Consequently, when the volume of the extraction solvent attains specific threshold, the need for flavonoid dissolution is largely met. Additional increments in solvent volume result in progressively smaller gains in flavonoid dissolution and elevate extraction expenses [49].

### 3.3. Response Surface Optimization of Total Flavonoid Extraction

To optimize the extraction process, BBD was utilized, with the experimental conditions derived from preliminary experiments. The extraction was carried out under the specific conditions: a solid–liquid ratio of 1:30, an extraction temperature of 50 °C, and an ultrasonic extraction time of 30 min. The molar ratio of urea to betaine (factor A), the water content in BU (factor B), and the ultrasonic power (factor C) were selected as the independent variables for optimizing the total flavonoid extraction. In the BBD, a total of seventeen experimental runs were conducted to optimize these three parameters. The design and results of the experiments are presented in Table 3.

The Box–Behnken design matrix and the ANOVA outcomes are summarized in Table 4 which was used to assess the polynomial regression model. The mathematical depiction of the connection linking total flavonoid content (TFC) and the independent factors A, B, and C is given by the subsequent regression formula:

*TFC* = +6.07 + 0.1434*A* + 0.0331*B* + 0.1026*C* − 0.1161*AB* + 0.0619*AC* − 0.0875*BC* − 0.3102*A*^2^ − 0.3368*B*^2^ − 0.2850*C*^2^

An analysis was conducted to assess the significance of the linear, quadratic, and interaction terms. The ‘lack of fit’ term had a non-significant *p*-value, whereas the ‘model’ term had a highly significant *p*-value (*p* < 0.0001). The model’s coefficient of determination (R^2^) was 0.9717, with an adjusted R^2^ of 0.9352, suggesting that the predicted TFC values were highly consistent with the experimental data and well-aligned with the objectives of this study [47]. Additionally, Table 4 reveals that the linear terms A and C, all quadratic terms (A^2^, B^2^, C^2^), and the interaction term AB were statistically significant (*p* < 0.05). In contrast, the linear terms B and the remaining interaction terms (AC, BC) showed no statistical significance (*p* > 0.05).

**Figure 4:**
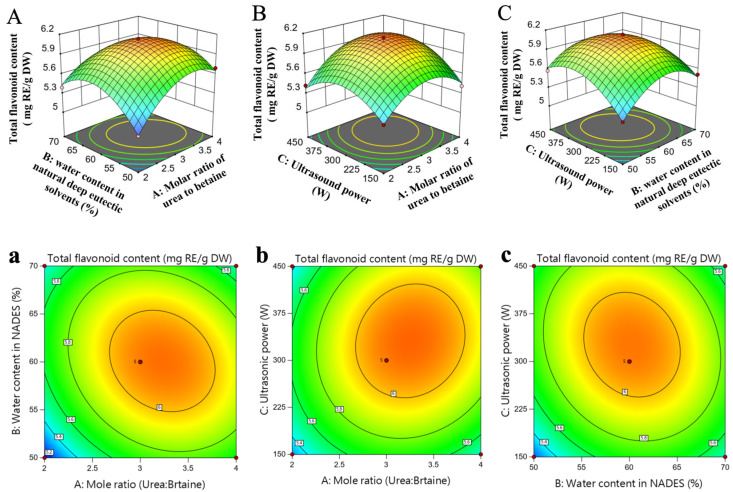
Response surface plots (**A**–**C**) and corresponding contour plots (**a**–**c**) of total flavonoid content extracted from wild blueberry using ultrasound-assisted betaine-urea extraction, affected by: the molar ratio of urea to betaine and water content in natural deep eutectic solvents (**A**,**a**); the molar ratio of urea to betaine and ultrasound power (**B**,**b**); and water content in natural deep eutectic solvents and ultrasound power (**C**,**c**). Different letters indicate significant differences (*p* < 0.05).

Figure 4 displays the 3D response surface plots and contour plots, presenting the variation in TFC in relation to the changes in the independent variables. Each diagram illustrates how two independent variables and their interaction affect TFC, with all other variables held constant. In general, when the surface in a 3D graph curve has a sharper incline, it suggests that the factor has a stronger impact. Conversely, if the contour plot takes on an oval shape, it implies a significant interaction exists between the two variables. Conversely, a contour plot that exhibits a circular shape suggests an absence of a significant interaction between the variables [47]. The 3D response surfaces exhibited steep slopes, and the contour lines were elliptical, indicating that the water content in NADESs exerted a significant effect on TFC extraction (Figure 4A,C). In Figure 4A,C, the interaction plots for A/B (urea-to-betaine molar ratio vs. water content) and B/C (urea-to-betaine molar ratio vs. ultrasound power) showed marked slopes with closely spaced, elliptical contour lines. This indicates strong interactions between the urea-to-betaine molar ratio and water content in NADES formulations, as well as between the urea-to-betaine molar ratio and ultrasound power. These interactions collectively and significantly influenced the TFC extraction yield. In contrast, the contour lines for the interaction between the urea-to-betaine molar ratio and ultrasound power (Figure 4B) were nearly circular, indicating that this interaction had no significant effect on TFC extraction. These findings are consistent with the analysis of variance results presented in Table 4.

Based on the regression model, the optimal parameters for extraction using UABU were determined as follows: a urea-to-betaine molar ratio of 3.256:1, a water content in NADESs of 59.774%, and an ultrasound power of 331.612 W, yielding a theoretical maximum value of 6.104 ± 0.037 mg_RE/g_DW. To validate these findings, verification experiments were carried out under adjusted practical conditions, including a urea-to-betaine molar ratio of 3.3:1, a water content of 60% in NADES, and an ultrasonic power of 330 W. Additionally, the ultrasonic-assisted extraction was performed with a solid-to-liquid ratio of 1:30, an extraction temperature of 50 °C, and an ultrasonic treatment duration of 30 min. The experimental TFC value obtained was 6.06 ± 0.024 mg_RE/g_DW, which closely matched the predicted value. This strong agreement between the experimental and predicted results indicates that the model has high accuracy and reliability with minimal error.

### 3.4. Utilizing LC-MS/MS-Based Untargeted Metabolomics for the Identification and In-Depth Analysis of Primary Flavonoid Compounds

**Figure 5:**
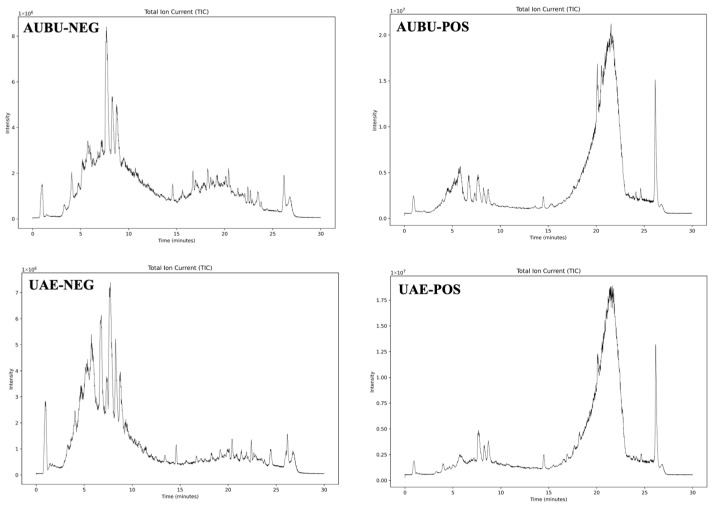
Total ion chromatogram of flavonoids extracted from ultrasound-assisted betaine-urea (AUBU) extractive and ultrasound-assisted 70% (*v*/*v*) ethanol (UAE) extractive in positive ion mode (POS) and negative ion mode (NEG).

**Figure 6:**
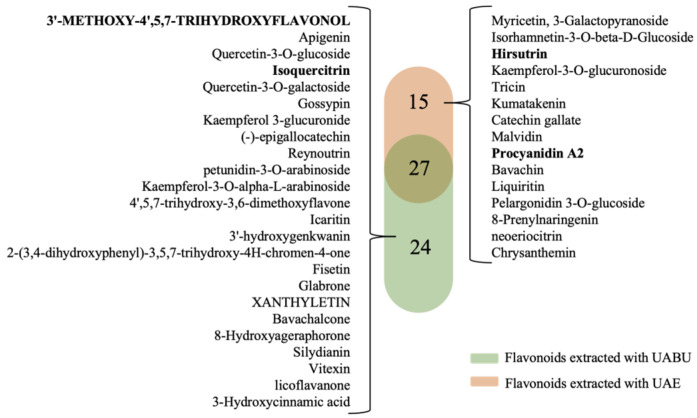
Venn diagram of differential components between flavonoids extracted with ultrasound-assisted betaine-urea and ultrasound-assisted 70% (*v*/*v*) ethanol.

The components extracted using UABU and UAE were analyzed based on Untargeted Metabolomics Analysis. This involved searching primary and secondary spectra against independently integrated databases from Metlin, MassBank, MoNA, and HMDB (version V6.0). It also combined accurate molecular weight and fragment ion information of the compounds. The total ion chromatograms in both positive and negative ion modes are illustrated in Figure 5. A total of 353 and 326 effective chemical components were identified using the two extraction methods, respectively. The identification results and the RC of flavonoids in the two solvent extracts, presented from high to low, are shown in Table 5 and Table 6. A total of 51 flavonoids were identified in the UABU extract, whereas 42 flavonoids were identified in the UAE extract. Twenty-seven flavonoid compounds were identified in both extraction methods. The components with higher content obtained from both extraction methods were generally similar, including oenin, isoquercetin, quercetin, quercetin-3-arabinoside, syringetin-3-O-glucoside, limocitrin, and laricitrin 3-galactoside. Furthermore, oenin emerged as the predominant flavonoid in both extraction systems. The RC of oenin in the UABU extract (20%) was significantly higher than in the UAE extract (5%). The RC of isorhamnetin-3-O-glucoside, myricetin 3-glucoside, and luteolin 4′-O-glucoside in the UABU extract was also higher compared to the UAE extract. Additionally, a total of 39 distinct flavonoids were identified in the two solvent extracts, with 24 flavonoids belonging to the UABU extract and the remaining 15 to the UAE extract, as shown in Figure 6. Among these, the levels of isoquercitrin (10%) and quercetin-3-O-glucoside (8.8%) were significantly higher in the UABU extract compared to the other flavonoids. These findings indicate that BU, as the NADES, is highly effective for extracting natural compounds. Furthermore, compared to a 70% (*v*/*v*) ethanol solution, BU enables the recovery of a more diverse range of bioactive components. The study carried out by Lei and colleagues [50] similarly revealed that the deep eutectic solvent (DES) system composed of choline chloride and lactic acid, when combined with ultrasonication, demonstrated superior performance compared to conventional solvent-based approaches in extracting total flavonoids from *Selaginella moellendorffii*. In comparison to earlier studies, the variety and yield of extracted compounds varied significantly across different NADESs, underscoring the need for further investigation into the underlying extraction mechanisms.

### 3.5. FTIR Spectra and DSC Analysis

**Figure 7:**
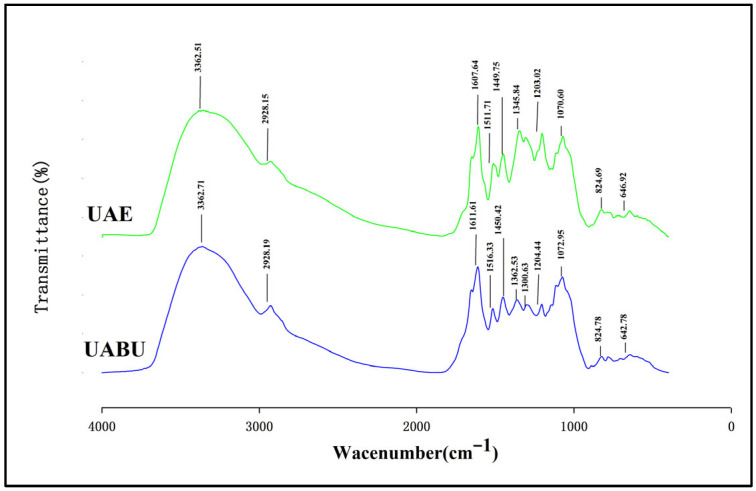
Effect of various extraction techniques on the FT-IR spectra of flavonoid extracts.

The structural alterations induced by UABU and UAE were assessed through FTIR analysis [51]. As illustrated in Figure 7, both UABU and UAE treatments resulted in phenolic profiles that were broadly comparable. Nevertheless, a notable discrepancy in phenolic composition was observed between the two methods. This variation may be due to the fact that NADES-based extraction depends on hydrogen bonding interactions between phenolic compounds and NADES, which enhance the solubility of these compounds [52]. Extracts from both treatments showed distinct peaks at approximately 3360, 2930, 1600, 1500, 1200, and 1070 cm^−1^. The FTIR absorption peak around 3360 cm^−1^ is associated with the O–H stretching vibrations that are characteristic of alcohols or phenolics [53], while the absorption band near 2930 cm^−1^ usually indicates the stretching of the aromatic C–H group in phenolic compounds [54]. Additionally, the absorption peaks at 1500 cm^−1^ and 1600 cm^−1^, respectively, suggest the existence of C–C double bonds and C=C bonds [55]. In addition, this research detected an ether group (C–O–C) with a band peak at 1200 cm^−1^, as well as a peak attributed to the C–O stretching vibration region at 1070 cm^−1^; this peak probably results from the aromatic ring vibrations of aromatic ether acids. Furthermore, the absorption peaks corresponding to O–H, C=C, and C–O–C in the UAE + ChCl-PG extract shifted to 1611 cm^−1^, 1516 cm^−1^, 1362 cm^−1^, and 1204 cm^−1^, respectively. This was mainly because of the increased strength of this peak in the extract obtained with UABU, consistent with findings from Bener et al. [56]. Therefore, UABU was found to be the suitable solvent.

**Figure 8:**
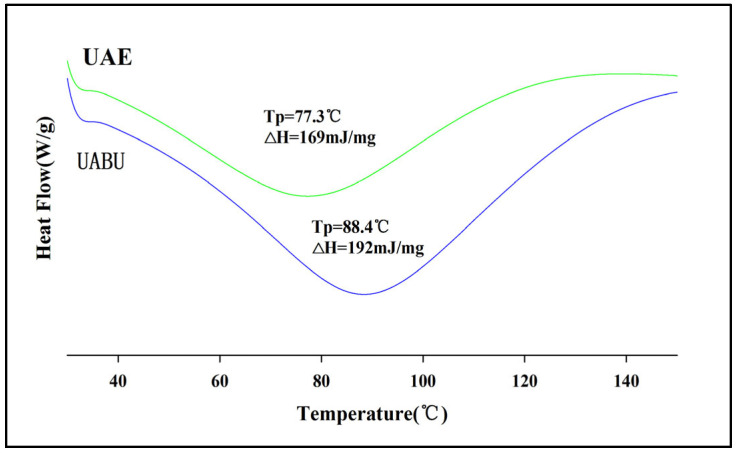
Effect of various extraction techniques on DSC thermograms of flavonoid extracts.

DSC serves as an effective analytical technique for assessing the thermal characteristics of phenolic compounds, providing insights into their composition and structural attributes [57]. As shown in Figure 8, the extracts obtained through the two distinct methods displayed a characteristic peak associated with the thermal decomposition of the extracted substances. Nevertheless, variations in the thermograms were observed due to the influence of NADES during the extraction process. The UABU sample exhibited a thermal denaturation temperature of 88.4 °C, which was 11.1 °C higher than that of the UAE sample (77.3 °C). Furthermore, the enthalpy change (ΔH) for the UABU sample was 192 mJ/mg, surpassing that of the UAE sample, which was 169 mJ/mg. This suggests that a greater amount of energy is required for the decomposition of the UABU sample, indicating a higher content of active groups susceptible to destruction upon heating [58]. These alterations, likely resulting from the synergistic effects induced by UABU, may stem from modifications in the composition and structure of the extracts. The thermal stability advantage thus conferred renders the UAUE extract more promising compared to its counterparts.

### 3.6. In Vitro Antioxidant Activities

**Figure 9:**
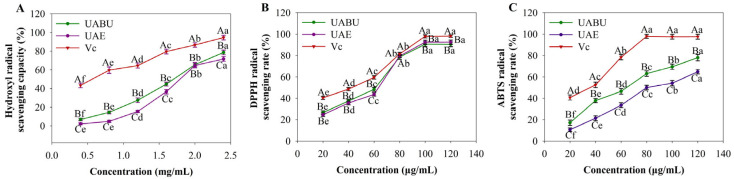
Antioxidant activities of the total flavonoids extracted from blueberries. (**A**) Hydroxyl radical scavenging activity; (**B**) DPPH radical scavenging activity; (**C**) ABTS radical scavenging activity. VC, vitamin C solution. Different uppercase letters indicate significant differences between groups (*p* < 0.05); different lowercase letters indicate significant differences within subgroups (*p* < 0.05). (Note: The “%” scavenging rates are calculated based on the initial radical concentrations in the assay system: DPPH•: 0.13 mM (prepared by dissolving DPPH powder in ethanol to an absorbance of 0.8–1.0 at 517 nm, corresponding to ~50 μg/mL, MW = 394.3 g/mol). ABTS•+: ~0.07 mM (generated by reacting 0.07 mM (NH_4_)_2_ABTS with 0.03 mM K_2_S_2_O_8_ in the dark for 12 h, then diluted to an absorbance of 0.7 ± 0.02 at 734 nm).

The antioxidant potential of flavonoid extracts in vitro is typically evaluated through their ability to scavenge free radicals. In this investigation, three key indicators, hydroxyl radical scavenging capacity (Figure 9A), DPPH radical scavenging rate (Figure 9B), and ABTS radical scavenging rate (Figure 9C), were selected to assess the antioxidant properties of the extracts. As depicted in Figure 9, within the proposed concentrations, the flavonoid extracts demonstrated antioxidant activities in a concentration-dependent response. The scavenging effects of the extracts significantly increased with higher flavonoid concentrations (*p* < 0.05). Moreover, the flavonoid extracts exhibited a higher scavenging rate for DPPH and ABTS radicals compared to hydroxyl radicals at the same concentration (1.0 mg/mL). The antioxidant performance of flavonoids extracted using UABU surpassed that of flavonoids obtained through UAE extraction, highlighting a direct correlation between flavonoid content and antioxidant efficacy. Flavonoids are equipped with hydroxyl groups, which function as electron donors; these groups interact with free radicals, resulting in the formation of stable intermediates and thereby putting an end to the chain reaction [50]. This mechanism agrees with the findings of Wu et al. [59], who reported a strong positive relationship between gingerol content and flavonoid antioxidant activity. One of the contributing factors is the lower volatility of NADESs compared to ethanol, which enhances flavonoid solubility and thereby further contributes to their antioxidant potential [60].

It is worth noting that NADES components, such as betaine, are essential biological molecules and are considered non-toxic [61]. Therefore, BU combined with ultrasonic extraction represents a promising and efficient approach for improving the antioxidant performance of blueberry-derived flavonoids.

### 3.7. Discussion

Although NADESs exhibit advantages such as biodegradability and low toxicity, they also present several challenges. The synthesis of NADESs can be relatively intricate and time-consuming, necessitating precise control over the molar ratios of components and reaction conditions. This may impede the large-scale production and widespread application of the NADES-based extraction method in industrial settings where cost-effectiveness and efficiency are of paramount importance. Furthermore, alterations in environmental conditions may induce changes in the physical and chemical properties of NADESs, which could potentially affect the extraction efficiency and reproducibility of target analytes [62]. Currently, there is a lack of well-established methods for achieving complete and effective recovery of NADESs from extraction mixtures, resulting in the loss of these solvents. Even when successfully recovered, the performance of NADESs in subsequent extraction cycles may deteriorate. For example, the binding of bioactive compounds to the components of NADESs can modify the solvent characteristics, leading to a decline in extraction efficiency [63]. Moreover, the recovery and reuse of NADESs entail additional costs and operational complexities. These include equipment (such as centrifuges and filters) and reagents (such as washing solvents) required for the recovery process, as well as the time and labor involved in the recovery and purification steps, all of which may reduce the overall process efficiency. In large-scale industrial environments, these factors may offset the potential economic benefits of using NADESs as green solvents. Therefore, further research is warranted to investigate the long-term stability of NADESs under different storage and operating conditions, as well as the number of reuse cycles of NADESs and the corresponding changes in their performance after recovery.

## 4. Conclusions

In the present study, by screening 22 NADESs solutions according to their impacts on the extraction yields of total flavonoids from blueberries, BU was identified as the optimal extractant. The maximum extraction yield of 6.06 ± 0.024 mg_RE/g_DW was achieved obtained under the following conditions: 60 wt% water content, 3.3:1 urea-to-betaine molar ratio, 330 W ultrasonic power, 1:30 g/mL solid-to-liquid ratio, 50 °C extraction temperature, and 30 min ultrasonic extraction time. The LC-MS/MS component analysis results demonstrate that UABU can more effectively extract flavonoids from blueberries. IR analysis revealed that the extract obtained using UABU exhibited an enhanced intensity of this characteristic peak, while DSC results demonstrated that this thermal stability advantage renders the UAUE extract more viable compared to other extracts.IR and DSC indicate that UAUE is a more suitable extraction solvent. Additionally, the flavonoids extracted by UABU showed stronger hydroxyl radical, DPPH, and ABTS radical scavenging activities than those obtained by UAE. In conclusion, compared to traditional organic solvents, NADESs are not only greener and more sustainable but also achieve higher TFC, richer flavonoid compositions, and superior antioxidant performance. This research established a green, environmentally friendly, cost-effective, and efficient extraction method for total flavonoids from wild blueberries, filling the existing research gap in the extraction of blueberry flavonoid compounds using NADESs. It provides a novel strategy for blueberry flavonoids extraction and feasible evidence for replacing traditional organic solvents with NADESs in natural product extraction.

## Figures and Tables

**Table 1:** Composition of natural deep eutectic solvents.

NADESs NO.	HBA	HBD	Molar Ratio	Abbreviation
NADES-1	Choline chloride	Sorbitol	1:1	ChSor
NADES-2		Ethylene glycol	1:1	ChEG
NADES-3		Propylene glycol	1:1	ChPG
NADES-4		Butanediol	1:1	ChBut
NADES-5		Citric acid	1:1	ChCA
NADES-6		Malic acid	1:1	ChMA
NADES-7		Oxalic acid	1:1	ChOA
NADES-8		Lactic acid	1:1	ChLA
NADES-9		Tartaric acid	1:1	ChTA
NADES-10		Urea	1:1	ChU
NADES-11		Proline	1:1	ChPro
NADES-12	Betaine	Sorbitol	1:1	BSor
NADES-13		Ethylene glycol	1:1	BEG
NADES-14		Propylene glycol	1:1	BPG
NADES-15		Butanediol	1:1	BBut
NADES-16		Citric acid	1:1	BCA
NADES-17		Malic acid	1:1	BMA
NADES-18		Oxalic acid	1:1	BOA
NADES-19		Lactic acid	1:1	BLA
NADES-20		Tartaric acid	1:1	BTA
NADES-21		Urea	1:1	BU
NADES-22		Proline	1:1	BPro

Note: the water content in all NADES was 30 wt%.

**Table 2:** Mobile phase elution gradient.

Time (min)	Flow-Rate (μL/min)	A (%)	B (%)
0.00	400	95	5
1.50	400	95	5
2.50	400	90	10
14.00	400	60	40
22.00	400	5	95
25.00	400	5	95
26.00	400	95	5
30.00	400	95	5

**Table 3:** Presents the Box–Behnken Design experimental setup, which incorporates diverse factor combinations and their corresponding levels to investigate total flavonoid content.

Run	Factor A	Factor B	Factor C	TFC
	A: Molar Ratio of Urea to Betaine	B: Water Content in NADESs (%)	C: Ultrasound Power (W)	Experimental Values (mg_RE/g_DW)
1	3	60	300	6.13
2	3	60	300	6.21
3	3	60	300	6.02
4	2	50	300	5.13
5	3	50	150	5.23
6	3	70	150	5.51
7	2	70	300	5.39
8	2	60	450	5.42
9	4	50	300	5.69
10	3	70	450	5.50
11	3	60	300	5.95
12	4	60	450	5.79
13	3	50	450	5.57
14	3	60	300	6.06
15	4	60	150	5.41
16	2	60	150	5.30
17	4	70	300	5.49

**Table 4:** ANOVA was performed to evaluate the linear, quadratic, and interaction effects among various factors.

Source	Sum of Squares	df	Mean Square	*F* Value	*p*-Value	
Model	1.73	9	0.1917	26.66	0.0001	significant
A: Molar ratio of urea to betaine	0.1645	1	0.1645	22.88	0.0020	
B: Water content in NADESs	0.0088	1	0.0088	1.22	0.3064	
C: Ultrasonic power	0.0843	1	0.0843	11.72	0.0111	
AB	0.0539	1	0.0539	7.49	0.0290	
AC	0.0153	1	0.0153	2.13	0.1877	
BC	0.0306	1	0.0306	4.26	0.0780	
A^2^	0.4051	1	0.4051	56.33	0.0001	
B^2^	0.4777	1	0.4777	66.44	<0.0001	
C^2^	0.3420	1	0.3420	47.57	0.0002	
Residual	0.0503	7	0.0072			
Lack of Fit	0.0106	3	0.0035	0.3541	0.7899	not significant
Pure Error	0.0398	4	0.0099			
Cor Total	1.78	16				
R^2^				0.9717		
Adjusted R^2^				0.9352		

**Table 5:** The blueberry flavonoids extracted with ultrasound-assisted betaine-urea tentatively characterized using LCMS/MS.

Flavonoid	RT (min)	Ion Mode	Formula	Precursor (*m*/*z*)	RC (%)
Oenin	5.8	[M+H]^+^	C_23_H_24_O_12_	493.1349	20.3
3′-METHOXY-4′,5,7-TRIHYDROXYFLAVONOL	18.6	[M+H]^−^	C_16_H_12_O_7_	315.0566	10.1
Isorhamnetin-3-O-glucoside	5.2	[M+H]^+^	C_22_H_22_O_12_	479.1201	9.9
Isoquercitrin	4.5	[M+H]^+^	C_21_H_20_O_12_	465.1058	8.8
Isoquercetin	7.6	[M+H]^+^	C_21_H_20_O_12_	465.103	3.4
Myricetin 3-glucoside	6.7	[M+H]^+^	C_21_H_20_O_13_	481.1002	3.3
luteolin 4′-O-glucoside	5.0	[M+H]^+^	C_21_H_20_O_11_	449.1081	2.5
Quercetin	7.6	[M+H]^+^	C_15_H_10_O_7_	303.052	2.1
Syringetin-3-O-glucoside	8.7	[M+H]^+^	C_23_H_24_O_13_	509.1299	1.8
Peonidin 3-O-glucoside	5.7	[M+H]^+^	C_22_H_22_O_11_	463.1252	1.8
Laricitrin 3-galactoside	7.8	[M+H]^+^	C_22_H_22_O_13_	495.1131	1.4
Quercetin-3-O-glucoside	3.6	[M+H]^+^	C_21_H_20_O_12_	465.105	1.3
Quercetin-3-Arabinoside	8.3	[M+H]^+^	C_20_H_18_O_11_	435.0935	1.2
Limocitrin	8.7	[M+H]^+^	C_17_H_14_O_8_	347.0782	1.2
Myricetin-3-Xyloside	7.3	[M+H]^+^	C_20_H_18_O_12_	451.0887	1.0
quercetin 3-O-glucuronide	7.8	[M+H]^+^	C_21_H_18_O_13_	479.0844	1.0
Quercetin-3-O-galactoside	1.0	[M+H]^+^	C_21_H_20_O_12_	465.1058	0.6
Reynoutrin	4.1	[M+H]^+^	C_20_H_18_O_11_	435.0934	0.6
4′,5,7-trihydroxy-3,6-dimethoxyflavone	5.8	[M+H]^+^	C_17_H_14_O_7_	331.0808	0.5
Kaempferol-3-O-alpha-L-arabinoside	5.3	[M+H]^+^	C_20_H_18_O_10_	419.0987	0.5
Isorhamnetin	5.2	[M+H]^+^	C_16_H_12_O_7_	317.0671	0.4
Myricetin	9.3	[M+H]^+^	C_15_H_10_O_8_	319.0463	0.4
Naringenin	18.9	[M+H]^−^	C_15_H_12_O_5_	271.0582	0.3
Quercetin-4′-O-glucoside	8.4	[M+H]^+^	C_21_H_20_O_12_	465.105	0.2
Apigenin	19.4	[M+H]^−^	C_15_H_10_O_5_	269.0435	0.2
Isorhamnetin 3-glucoside	9.4	[M+H]^+^	C_22_H_22_O_12_	479.121	0.1
Fisetin	5.0	[M+H]^+^	C_15_H_10_O_6_	287.0542	0.1
petunidin-3-O-arabinoside	9.3	[M+H]^+^	C_21_H_20_O_11_	449.1072	0.1
Vitexin	6.7	[M+H]^+^	C_21_H_20_O_10_	433.1147	0.1

**Table 6:** The blueberry flavonoids extracted with ultrasound-assisted 70% ethanol tentatively characterized using LCMS/MS.

Flavonoid	RT (min)	Ion Mode	Formula	Precursor (*m*/*z*)	RC (%)
Isoquercetin	7.6	[M+H]^+^	C_21_H_20_O_12_	465.1031	8.0
Hirsutrin	7.6	[M+H]^+^	C_21_H_20_O_12_	465.1121	5.5
Oenin	5.9	[M+H]^+^	C_23_H_24_O_12_	493.1356	5.2
Syringetin-3-O-glucoside	8.7	[M+H]^+^	C_23_H_24_O_13_	509.1307	4.5
Limocitrin	8.7	[M+H]^+^	C_17_H_14_O_8_	347.0788	3.0
Laricitrin 3-galactoside	7.8	[M+H]^+^	C_22_H_22_O_13_	495.1139	2.9
Quercetin-3-Arabinoside	8.3	[M+H]^+^	C_20_H_18_O11	435.0943	2.8
Quercetin	8.3	[M+H]^+^	C_15_H_10_O_7_	303.0503	2.6
quercetin 3-O-glucuronide	7.8	[M+H]^+^	C_21_H_18_O_13_	479.0821	1.2
Procyanidin A2	7.2	[M+H]^+^	C_30_H_24_O_12_	577.1346	1.0
Isorhamnetin-3-O-beta-D-Glucoside	8.6	[M+H]^+^	C_22_H_22_O_12_	479.1183	0.6
Quercetin-4′-O-glucoside	8.4	[M+H]^+^	C_21_H_20_O_12_	465.103	0.5
Isorhamnetin	8.6	[M+H]^+^	C_16_H_12_O7	317.0679	0.5
Isorhamnetin 3-glucoside	9.4	[M+H]^+^	C_22_H_22_O_12_	479.1207	0.4
Peonidin 3-O-glucoside	5.8	[M+H]^+^	C_22_H_22_O_11_	463.1252	0.3
Myricetin 3-glucoside	6.7	[M+H]^+^	C_21_H_20_O_13_	463.1252	0.2
Chrysanthemin	9.3	[M+H]^+^	C_21_H_20_O_11_	449.1079	0.2
Myricetin, 3-Galactopyranoside	6.7	[M+H]^+^	C_21_H_20_O_13_	481.1073	0.1
Malvidin	5.8	[M+H]^+^	C_17_H_14_O_7_	331.0808	0.1
Myricetin	7.4	[M+H]^+^	C_15_H_10_O_8_	319.0471	0.1
Myricetin-3-Xyloside	7.4	[M+H]^+^	C_20_H_18_O_12_	451.0871	0.1
Kaempferol-3-O-glucuronoside	8.6	[M+H]^+^	C_21_H_18_O_12_	463.0876	0.1

## Data Availability

The original contributions presented in the study are included in the article, further inquiries can be directed to the corresponding author.
